# LncRNA DLGAP1-AS2 regulates miR-503/cyclin D1 to promote cell proliferation in non-small cell lung cancer

**DOI:** 10.1186/s12890-021-01633-0

**Published:** 2021-08-28

**Authors:** Lu Wang, Lei Tang, Tengfei Ge, Feng Zhu, Dan Liu, Hua Guo, Peng Qian, Ning Xu

**Affiliations:** Department of Thoracic Surgery, Anhui Chest Hospital, No. 397 Jixi Road, Shushan District, Hefei City, Anhui Province 230022 People’s Republic of China

**Keywords:** Non-small cell lung cancer, DLGAP1-AS2, miR-503, Cyclin D1

## Abstract

**Background:**

LncRNA DLGAP1-AS2 plays an oncogenic role in glioma, while its role in other cancers is unknown. This study aimed to study the role of DLGAP1-AS2 in non-small cell lung cancer (NSCLC).

**Methods:**

Expression of DLGAP1-AS2 in NSCLC and paired non-tumor tissues from 64 NSCLC patients and the prognostic value of DLGAP1-AS2 for NSCLC were analyzed by performing a 5-year follow-up study. The interaction between DLGAP1-AS2 and miR-503 was confirmed by dual luciferase reporter assay, and their relationship was explored in NSCLC cells transfected with DLGAP1-AS2 expression vector or miR-503 mimic. The roles of DLGAP1-AS2 and miR-503 in regulating cyclin D1 expression were analyzed by RT-qPCR and Western blot. Cell proliferation was analyzed by CCK-8 assay.

**Results:**

DLGAP1-AS2 was upregulated in NSCLC and predicted poor survival. Interaction between DLGAP1-AS2 and miR-503 was confirmed by dual luciferase activity assay. Overexpression experiments showed that DLGAP1-AS2 and miR-503 overexpression failed to significantly affect the expression of each other. Interestingly, DLGAP1-AS2 overexpression upregulated cyclin D1, a target of miR-503, increased cell proliferation and reduced the effects of miR-503 overexpression on cyclin D1 expression and cell proliferation.

**Conclusions:**

DLGAP1-AS2 may regulate miR-503/cyclin D1 to promote cell proliferation in NSCLC.

**Supplementary Information:**

The online version contains supplementary material available at 10.1186/s12890-021-01633-0.

## Background

Lung cancer is the major cause of cancer deaths among both females and males worldwide [1, 2]. It accounted for about 11.6% of all new cancer cases and caused 18.4% of all cancer deaths in 2018 [3]. More than half of lung cancer patients diagnosed at early stages can survive more than 5 years [4]. However, tumor metastasis to distant organs, such as the brain, bones, and liver, is common in lung cancer patients [5]. Despite the efforts made to treat metastatic lung cancer patients, only fewer than 5% of them can survive more than 5 years [5]. Smoking is the major contributor to the development of lung cancer, while lung cancer also affects never-smokers [6, 7], suggesting the involvement of other internal and/or environmental factors.

During the past several decades, studies of the molecular pathology of lung cancer have revealed multiple signaling pathways with crucial functions in the growth and metastasis of lung cancer [8]. With our understanding of the molecular mechanisms of lung cancer increasing, novel therapies, such as targeted therapy that can be applied to suppress tumor growth and metastasis, have been developed [9, 10]. However, effective targets for lung cancer remain lacking. LncRNAs and miRNAs are noncoding RNAs (ncRNAs) that participate in cancer by regulating the expression of related genes [11, 12], suggesting that lncRNAs and miRNAs are promising targets for cancer therapy. In a recent study, DLGAP1-AS2 was reported to promote the development of glioma [13]. We performed bioinformatics analysis and predicted the potential interaction between DLGAP1-AS2 and miR-503, targeting cyclin D1 to suppress tumor growth [14]. This study aimed to explore the interactions among DLGAP1-AS2, miR-503, and cyclin D1 in non-small cell lung cancer (NSCLC).

## Methods

### Patients and follow-up

The study was approved by the Ethics Committee of Anhui Chest Hospital (No. Ach#965) and enrolled a total of 64 NSCLC patients (42 males and 22 females) between May 2013 and May 2015. These patients were between the age of 46 to 68 years, with a mean of 57.6 ± 6.6 years. Patients who (1) had other clinical disorders or recurrent NSCLC, (2) accepted previous treatment, and (3) died of diseases other than NSCLC were excluded from the study. These 64 patients were classified into AJCC stage I or II (n = 26) and stage III or IV (n = 38). All patients were visited monthly for a total of 5 years to record their survival status. The written informed consent was obtained from all patients. The clinicopathologic parameters of 64 NSCLC patients were shown in Table 1.

**Table 1:** Correlation of DLGAP1-AS2 expression with clinicopathologic parameters in 64 NSCLC patients

Clinicopathologic parameter	Total (n = 64)	DLGAP1-AS2 expression level		*P*-value
		Lower (n = 33)	Higher (n = 31)	
*Age (years)*				0.321
≤ 57	36	15	21	
> 57	28	16	12	
*Sex*				0.756
Male	39	22	17	
Female	25	12	13	
*AJCC stage*				0.360
I/II	26	18	8	
III/IV	38	10	28	
*Histological grade*				0.839
Well and middle	34	16	18	
Low	30	15	15	
*Tumor diameter*				0.004**
T ≤ 5 cm	31	21	10	
T > 5 cm	33	9	24	
*Lymph node*				0.213
Negative	34	25	9	
Positive	30	8	22	

### NSCLC tissues and cells

Before therapy, NSCLC tissues and their paired non-tumor tissues were collected from each patient using fine-needle aspiration, confirmed using histopathological analysis, and stored in liquid nitrogen for subsequent analyses.

H2170 NSCLC cell line (ATCC® CRL-5928™) were from ATCC (USA) and cultured in RPMI medium supplemented with 10% FBS in a 5% CO_2_ incubator at 37 °C with 95% humidity.

### Transient transfections and dual luciferase reporter assay

DLGAP1-AS2 or cyclin D1 expression vectors were constructed using pcDNA3.1 as the backbone vector (Invitrogen). Negative control (NC) miRNA and mimic of miR-503 were purchased from Sigma-Aldrich. H2170 cells were transfected with 1 µg DLGAP1-AS2 or 40 nM miR-503 mimic using Lipofectamine 2000 (Invitrogen). Cells transfected with either empty vector or NC miRNA were used as the NC. In addition, non-transfected cells were served as the control (C).

DLGAP1-AS2 luciferase reporter vector was constructed with pGL3 luciferase vector as the backbone (Promega) and co-transfected with miR-503 mimic (miR-503 group) or + NC miRNA (NC group) into H2170 cells. At 48 h of post-transfection, luciferase activity assay was performed to explore the interaction between DLGAP1-AS2 and miR-503 mimic.

### RNA isolation

Total RNA was isolated from paired tissue samples and H2170 cells using Ribozol reagent (Invitrogen). After being treated with DNase I (Invitrogen) for 100 min at 37 °C to completely remove genomic DNAs, RNA integrity was examined by electrophoresis on 6% urea-PAGE gels. The purity of RNA samples was reflected by OD260/280 ratios.

### RT-qPCR

RNA samples were subjected to reverse transcriptions (RTs) using the SS-IV-RT system (Invitrogen) to prepare cDNA samples. The levels of DLGAP1-AS2 and cyclin D1 mRNAs were determined using qPCRs with 18S rRNA as an internal control using SYBR Green Master Mix (Bio-Rad). MiR-503 level was determined using All-in-One™ miRNA qRT-PCR Reagent Kit (Genecopoeia) with U6 as the internal control.

Three technical replicates were included in each experiment. Ct values were normalized using the method of 2^−ΔΔCt^ [15, 16]. The primers used in qPCR were DLGAP1-AS2 forward 5′-TTCCTGTCTTTCAGGATGAATGCC-3′ and reverse 5′-TGGTAGC CTGTGGCGAGTTGAA-3′; cyclin D1 forward 5′-CGAGGAGCTGCTGCAAATGG-3′ and reverse 5′-CAGAGGGCAACGAAGGTCTG-3′; 18S rRNA forward 5′-GAG AAACGGCTACCACATCCA-3′ and reverse 5′-CGTGCCATCCCAAAGTCCAAC-3′; miR-503 forward 5′-CCTATTTCCCATGATTCCTTCATA-3′ and reverse 5′-GTAATA CGGTTATCCACGCG-3′; and U6 forward 5′-CTCGCTTCGGCAGCACA-3′ and reverse 5′-AACGCTTCACGAATTTGCGT-3′.

### Western blot

Proteins were extracted from H2170 cells using RIPA solution (Invitrogen). After quantification using BCA assay (Invitrogen), the same amount of proteins were denatured at 95 °C for 10 min, separated by electrophoresis on SDS-PAGE gels, and transferred onto PVDF membranes. The membranes were incubated with 5% fat-free milk (PBS) at room temperature for 2 h before incubation with primary antibodies against cyclin D1 (ab194972, Abcam) and GAPDH (ab9845, Abcam) for 16 h at 4 °C. After that, the membranes were incubated with HRP IgG secondary antibody (ab6721, Abcam) for 3 h at 25 °C. Protein signals were developed using ECL (Invitrogen), detected, analyzed, and normalized to the control using Quantity One software.

### Cell Counting Kit-8 (CCK-8) assay

H2170 cells with transfections were transferred to a 96-well cell culture plate with 0.1 ml medium containing 3000 cells per well and cultured at 37 °C. OD values at 450 nm were measured every 24 h for a total of 4 days at 2 h after incubation with 10% CCK-8 solution.

### Cell colony formation assay

H2170 cells (1 × 10^3^ cells/well) transfected with indicated vectors were plated in 6-well plates and cultured at 37 °C in a humidified incubator with 5% CO_2_ for 14 days. Colonies were fixed using 100% methanol, stained with 0.1% crystal violet, counted, and analyzed.

### Statistical analysis

DLGAP1-AS2 levels in paired tissues were expressed as the average of three technical replicates and compared by paired t test. Luciferase activity was expressed as Mean ± SD value and compared by unpaired t-test. Data from multiple transfection groups was expressed as Mean ± SD value of three biological replicates and compared using ANOVA Tukey’s test. To perform survival analysis, the 64 patients were divided into high and low DLGAP1-AS2 level groups (n = 32) with the median value as the cutoff. Survival curves were plotted for both groups and compared using log-rank test. *p* < 0.05 was deemed statistically significant.

## Results

### DLGAP1-AS2 was overexpressed in NSCLC and correlated with the poor survival of NSCLC patients

**Fig. 1:**
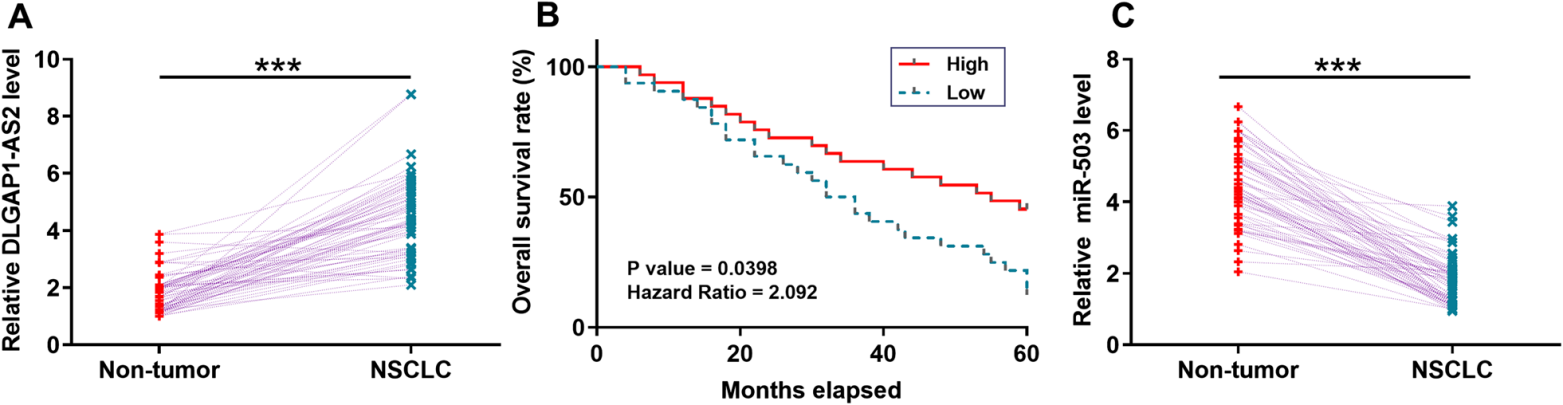
DLGAP1-AS2 was overexpressed in NSCLC and correlated with the poor survival of NSCLC patients. Expression of DLGAP1-AS2 and miR-503 in NSCLC tissues and their paired non-tumor tissues collected from 64 patients was determined by RT-qPCR. DLGAP1-AS2 levels in the paired tissues were expressed as the average of three technical replicates (**A**). ***p < 0.001. For survival analysis, the 64 patients were divided into high and low DLGAP1-AS2 level groups (n = 32). Survival curves were plotted for both groups and compared using the log-rank test (**B**). MiR-503 expression levels in the paired tissues were expressed as the average of three technical replicates (**C**). ***p < 0.001

DLGAP1-AS2 expression in NSCLC tissues and their paired non-tumor tissues collected from 64 patients was determined by RT-qPCR. DLGAP1-AS2 was significantly overexpressed in NSCLC tissues compared to the paired non-tumor tissues (Fig. 1A, *p* < 0.05). Survival curve analysis showed that the overall survival rate of NSCLC patients in the high DLGAP1-AS2 level group was significantly lower than that in the low DLGAP1-AS2 level group (Fig. 1B). Therefore, DLGAP1-AS2 overexpression in NSCLC was correlated with the poor survival of NSCLC patients. In addition, miR-503 was significantly downregulated in NSCLC tissues compared to the paired non-tumor tissues (Fig. 1C, *p* < 0.05).

### DLGAP1-AS2 and miR-503 directly interacted with each other

**Fig. 2:**
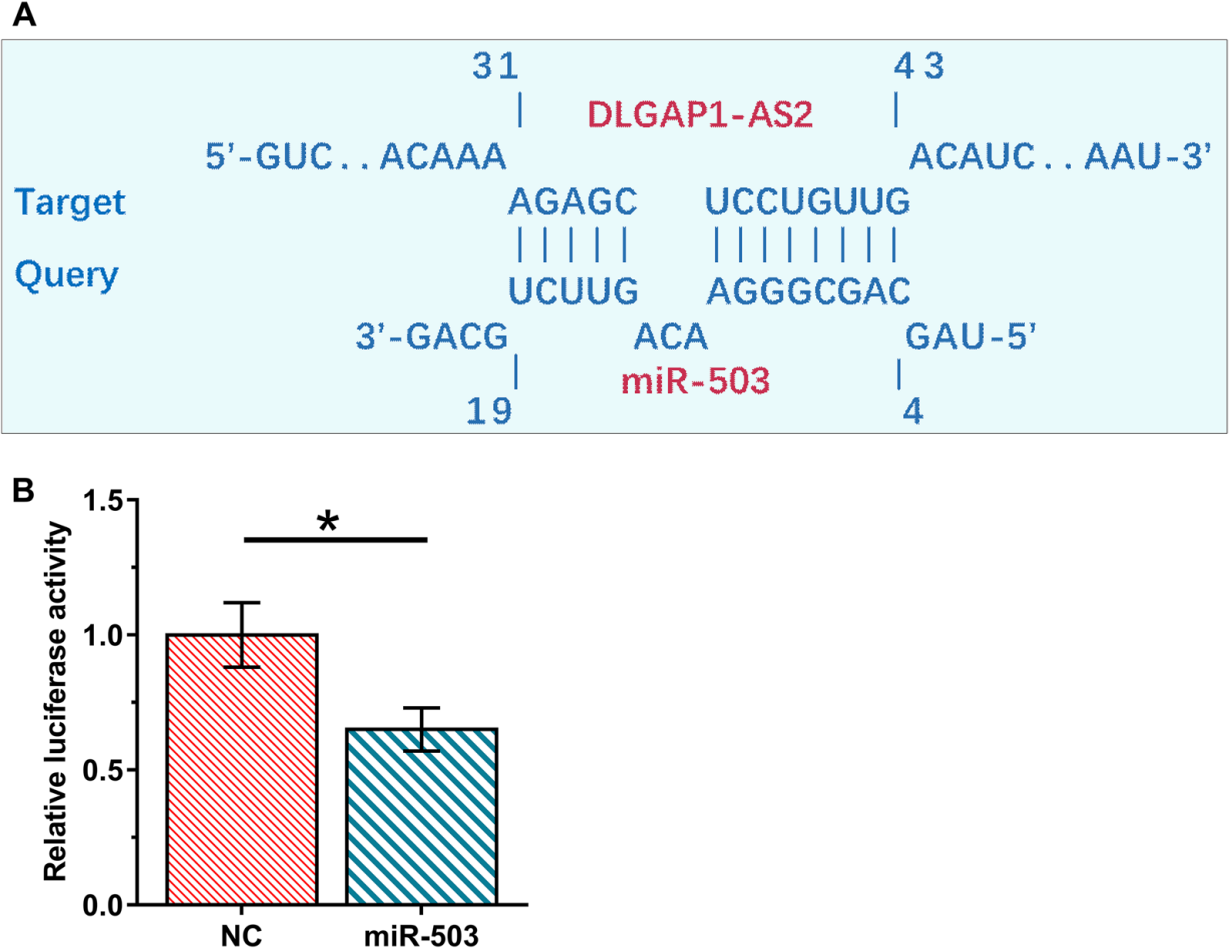
DLGAP1-AS2 and miR-503 directly interacted with each other. The interaction between DLGAP1-AS2 and miR-503 was predicted by IntaRNA 2.0 (**A**). For dual-luciferase reporter assay, DLGAP1-AS2 luciferase vector + miR-503 mimic (miR-503 group) or DLGAP1-AS2 luciferase vector + NC miRNA (NC group) was co-transfected into H2170 cells using Lipofectamine 2000. Luciferase activity was measured at 48 h of post-transfection (**B**)

The interaction between DLGAP1-AS2 and miR-503 was predicted by IntaRNA 2.0. It was observed that DLGAP1-AS2 and miR-503 could form multiple base pairings (Fig. 2A). Dual-luciferase reporter assay analysis showed that the luciferase activity was significantly lower in the miR-503 group compared to the NC group (Fig. 2B, *p* < 0.05), suggesting a direct interaction between them.

### Overexpression of DLGAP1-AS2 and miR-503 failed to regulate the expression of each other

**Fig. 3:**
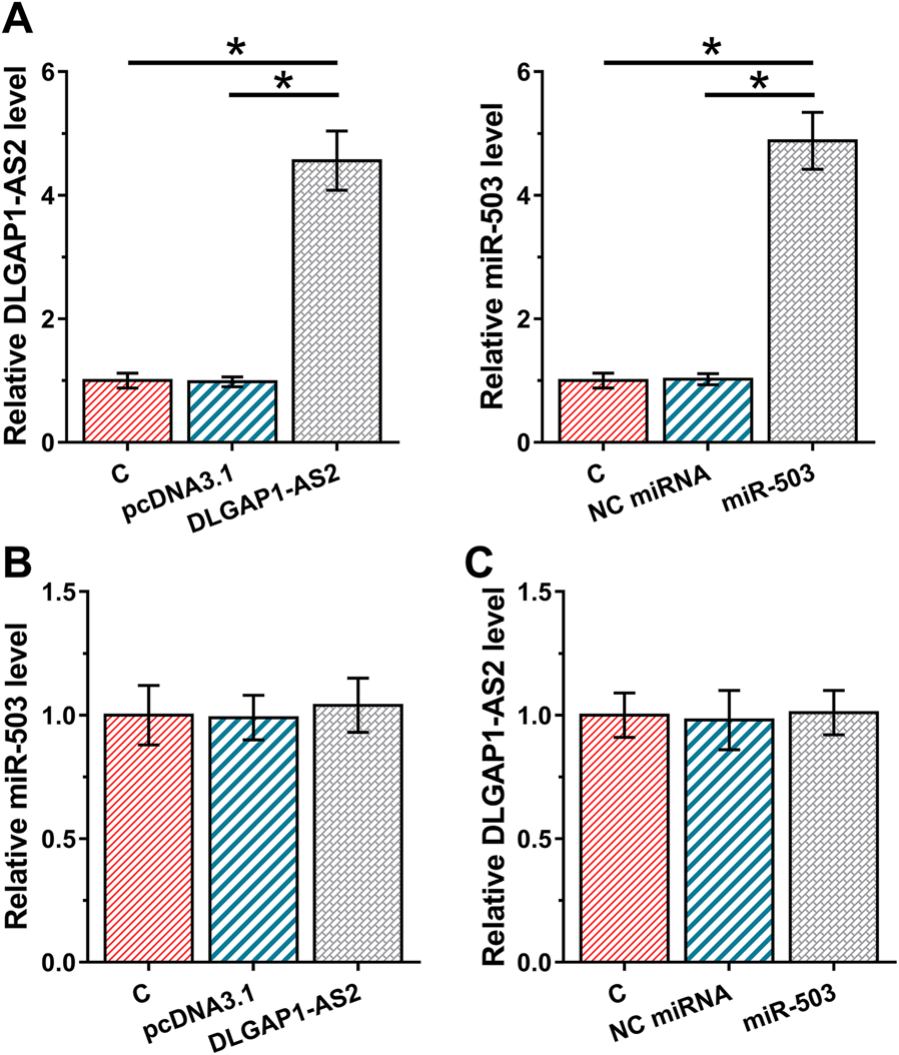
Overexpression of DLGAP1-AS2 and miR-503 failed to regulate the expression of each other. To further explore the interaction between DLGAP1-AS2 and miR-503, H2170 cells were transfected with DLGAP1-AS2 expression vector or miR-503 mimic, followed by confirmation of the transfections by RT-qPCR (**A**). In addition, the effects of DLGAP1-AS2 overexpression on miR-503 (**B**) and the effects of miR-503 overexpression on DLGAP1-AS2 (**C**) were analyzed by RT-qPCR. Mean ± SD values of three biological replicates were used to express data of multiple transfection groups. *p < 0.05

To further explore the interaction between DLGAP1-AS2 and miR-503, H2170 cells were transfected with DLGAP1-AS2 expression vector or miR-503 mimic, followed by confirmation of the transfections by RT-qPCR (Fig. 3A, *p* < 0.05). It was observed that DLGAP1-AS2 overexpression failed to significantly alter miR-503 expression (Fig. 3B). Moreover, miR-503 overexpression also showed no significant effects on DLGAP1-AS2 expression (Fig. 3C). Therefore, DLGAP1-AS2 is unlikely a target of miR-503 and could act as an endogenous sponge of miR-503.

### DLGAP1-AS2 overexpression increased the expression of cyclin D1, a target of miR-503

**Fig. 4:**
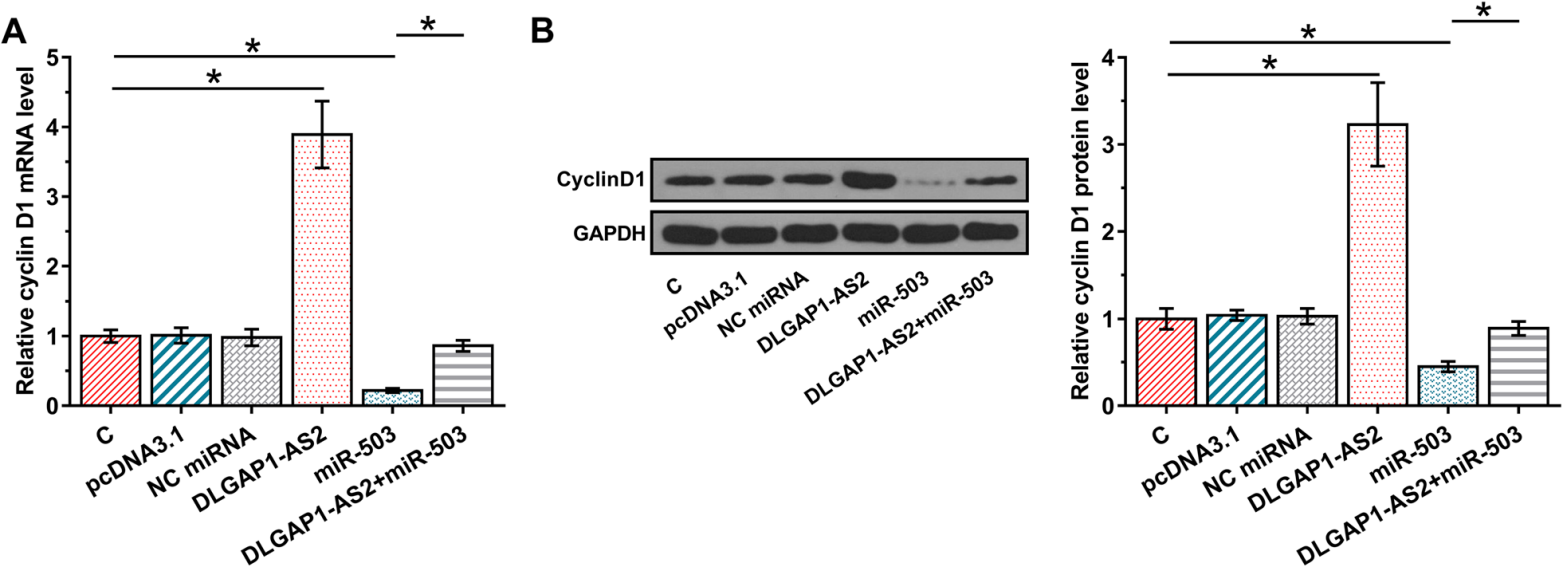
DLGAP1-AS2 overexpression increased the expression of cyclin D1, a target of miR-503. To test possibility that DLGAP1-AS2 acts as an endogenous sponge of miR-503, the effects of DLGAP1-AS2 and miR-503 overexpression on cyclin D1 expression were explored by RT-qPCR (**A**) and Western blot (**B**). Mean ± SD values of three biological replicates were used to express data of multiple transfection groups. *p < 0.05

To explore this possibility that DLGAP1-AS2 acts as an endogenous sponge of miR-503, the effects of DLGAP1-AS2 and miR-503 overexpression on cyclin D1 expression were explored by RT-qPCR (Fig. 4A) and Western blot (Fig. 4B). DLGAP1-AS2 overexpression increased cyclin D1 expression, while the transient transfection of miR-503 mimic decreased cyclin D1 expression compared with the NC miRNA (*p* < 0.05). Similarly, as shown as in Fig. S1, the cyclin D1 were knockdown and overexpression using cyclin D1-siRNA and cyclin D1 overexpression vector, respectively. The effects induced by DLGAP1-AS2 overexpression on cyclin D1 expression was reversed by transfecting cyclin D1-siRNA (*p* < 0.05). Moreover, cyclin D1 expression was downregulated in cells transfected with miR-503 mimics, and the effects induced by miR-503 overexpression on cyclin D1 expression was reversed by transfecting miR-503 mimic plus pcDNA3.1-DLGAP1-AS2 (Fig. 4B, *p* < 0.05). In addition, cyclin D1 expression was upregulated in cells transfected with miR-503 inhibitor, and the effects induced by DLGAP1-AS2 overexpression on cyclin D1 expression was reversed by transfecting miR-503 inhibitor (Additional file 1: Fig. S1, *p* < 0.05).

### DLGAP1-AS2/miR-503/cyclin D1 pathway regulated H2170 cell proliferation

**Fig. 5:**
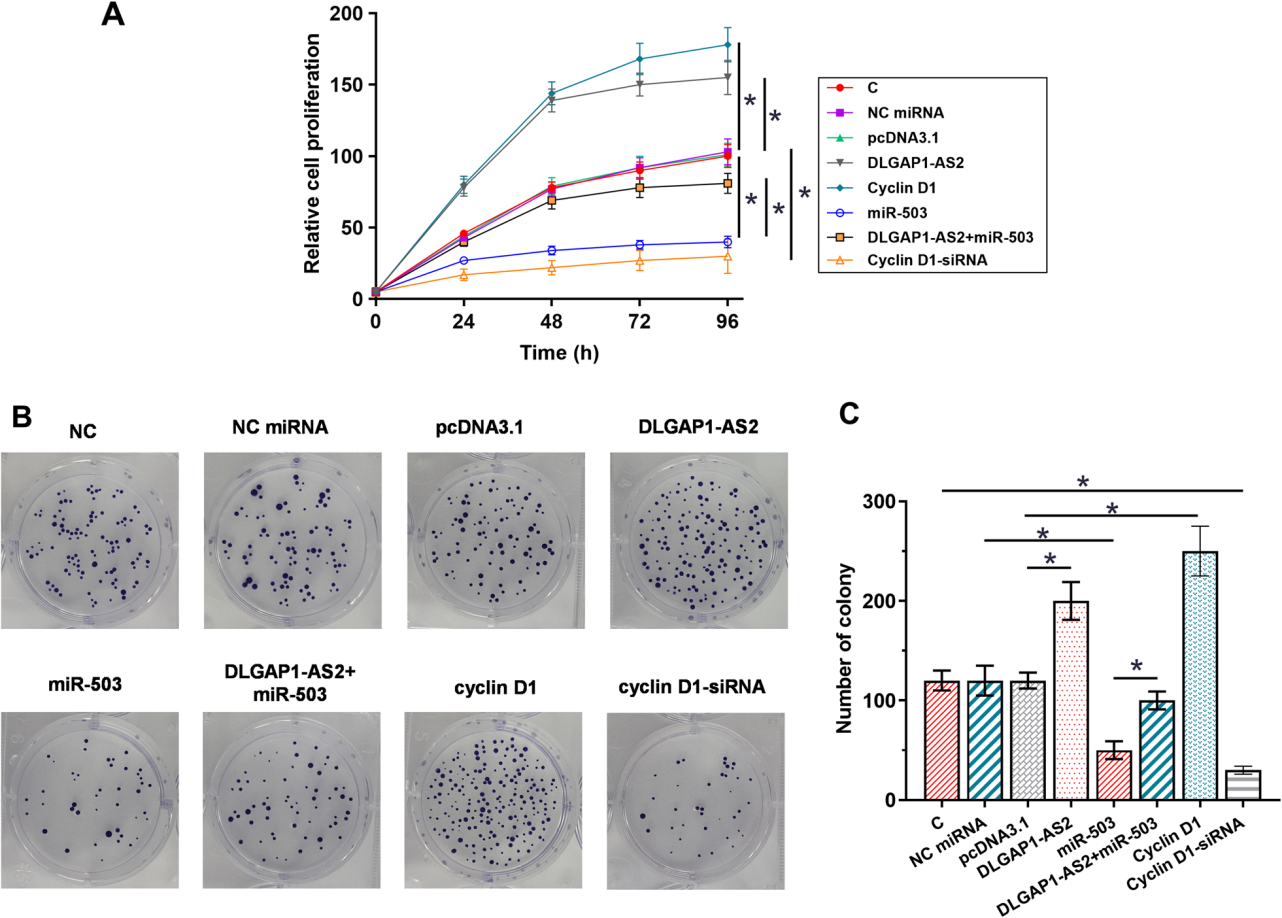
DLGAP1-AS2 /miR-503/cyclin D1 pathway regulated the proliferation of H2170 cells. The effects of the overexpression of DLGAP1-AS2, miR-503, and cyclin D1 on H2170 cell proliferation were analyzed by CCK-8 assay (**A**). Cell colony formation was shown at 14 days after transfection with indicated vector in H2170 cells (**B**, **C**). Mean ± SD values of three biological replicates were used to express data of multiple transfection groups. *p < 0.05

The effects of overexpression of DLGAP1-AS2, miR-503, and cyclin D1 on the H2170 cell proliferation were analyzed by CCK-8 and cell colony formation assays. As shown in Fig. 5A, overexpression of DLGAP1-AS2 or cyclin D1 increased cell proliferation compared with the empty vector (*p* < 0.05). Moreover, transient transfection of miR-503 mimic and cyclin D1-siRNA decreased H2170 cell proliferation compared with NC miRNA and untreated control, respectively (*p* < 0.05). Furthermore, the effects induced by miR-503 overexpression were reversed by transfecting miR-503 mimic plus pcDNA3.1-DLGAP1-AS2 (*p* < 0.05). Consistently, As shown in Fig. 5B,C, cell colony formation assays showed that miR-503 overexpression resulted in fewer and smaller colonies compared to the NC miRNA, and the effects were reversed by transfecting miR-503 mimic plus pcDNA3.1-DLGAP1-AS2 groups (*p* < 0.05). These results suggested that DLGAP1-AS2 overexpression reduced the effects of miR-503 overexpression on cell proliferation.

## Discussion

This study explored the interplay between DLGAP1-AS2 and the axis of miR-503/cyclin D1 in NSCLC. The results showed that DLGAP1-AS2 was overexpressed in NSCLC and promoted NSCLC cell proliferation by upregulating cyclin D1 by sponging miR-503.

The functionality of DLGAP1-AS2 has only been analyzed in glioma [13]. It was observed that DLGAP1-AS2 was overexpressed in glioma and upregulated YAP1 to promote glioma cell proliferation and metastasis [13]. However, the involvement of DLGAP1-AS2 in other cancers remains unclear. In this study, we showed that DLGAP1-AS2 was significantly overexpressed in NSCLC, and DLGAP1-AS2 overexpression increased the proliferation rate of NSCLC cells, indicating DLGAP1-AS2 overexpression in NSCLC may play oncogenic roles by enhancing cancer cell proliferation.

Despite the efforts made on NSCLC treatment, the overall survival of NSCLC patients remains poor [17, 18]. We found that high DLGAP1-AS2 expression levels were closely correlated with the poor survival of NSCLC patients. Therefore, measuring DLGAP1-AS2 expression levels in cancer tissues might provide guidance for developing personalized therapy, thereby improving patients’ survival. However, clinical trials are needed to verify our hypothesis.

MiR-503 plays different roles in different types of cancers [14, 19]. For instance, miR-503 is overexpressed in colorectal cancer and promotes cancer progression [19]. Cyclin D1, a target of miR-503 plays a critical role in various cancers. Jiang et al. found that miR-503 downregulation promotes proliferation, migration, and invasion of esophageal squamous cell carcinoma (ESCC) cells by targeting cyclin D1 [14]; Long et al. found that miR-503 inhibits human breast cancer cell proliferation by suppressing cyclin D1 expression (20); Xu et al. [21] found that miR-503 suppresses endometrioid endometrial cancer cell proliferation and cycle progression by negatively regulating cyclin D1. However, the role of miR-503 by targeting cyclin D1 is unclear in NSCLC. Therefore, in this study, we chose cyclin D1 as a potential target and showed that miR-503 might also target cyclin D1 in NSCLC cells to decrease cell proliferation. We found DLGAP1-AS2 directly interacts with miR-503 to regulate the cyclin D1 expression but has no effect on miR-503 expression. A similar phenomenon has been found in other lncRNAs. Yang et al. [22] showed that lncRNA MIR31HG functions as a miR-193b sponge but has no significant effects on miR-193b level following MIR31HG knockdown or overexpression. Yang et al. also showed that miR-193b overexpression suppresses MIR31HG expression and function, suggesting that MIR31HG is a target of miR-193b. However, in our study, miR-503 overexpression has no effect on DLGAP1-AS2 expression, suggesting that DLGAP1-AS2 is unlikely a target of miR-503. Therefore, miR503 overexpression inhibits H2170 cell proliferation by downregulating cyclin D1 expression, and DLGAP1-AS2 overexpression may act as a 'sponge' by competing for miR-503 binding to upregulate cyclin D1 expression, thereby promoting H2170 cell proliferation. Overall, our study showed that DLGAP1-AS2 overexpression reduces the effects of miR-503 overexpression on cyclin D1 expression and cell proliferation. Therefore, DLGAP1-AS2 may sponge miR-503 in NSCLC.

## Conclusion

DLGAP1-AS2 is overexpressed in NSCLC and might upregulate cyclin D1 by sponging miR-503 to promote NSCLC cell proliferation.

## Supplementary Information

**Additional file 1: Figure S1.** The relative cyclin D1 protein level in H2170 cells. To further reveal that DLGAP1-AS2 overexpression increased cyclin D1 expression, H2170 cells were transfected with miR-503 inhibitor and cyclin D1-siRNA vectors, and cyclin D1 expression was determined by Western blot. Mean ± SD values of three biological replicates were used to express data of multiple transfection groups. *p < 0.05.

## Data Availability

The data supporting the findings of this study are available on request from the corresponding author: Ning Xu, Department of Thoracic Surgery, Anhui Chest Hospital, No. 397 Jixi Road, Shushan District, Hefei City, Anhui Province, 230022, P. R. China. Email: NingXuHefei@163.com. Some data are not publicly available due to containing information that could compromise the privacy of research participants.

## Abbreviations

NSCLC: Non-small cell lung cancer
ncRNAs: Noncoding RNAs
ESCC: Esophageal squamous cell carcinoma
NC: Negative control
C: Control
CCK-8: Cell Counting Kit-8

## References

[1] Adjei AA (2019). Lung cancer worldwide. J Thoracic Oncol: Off Publ Int Assoc Study Lung Cancer.

[2] Barta JA, Powell CA, Wisnivesky JP (2019). Global epidemiology of lung cancer. Ann Glob Health.

[3] Bray F, Ferlay J, Soerjomataram I, Siegel RL, Torre LA, Jemal A (2018). Global cancer statistics 2018: GLOBOCAN estimates of incidence and mortality worldwide for 36 cancers in 185 countries. CA: Cancer J Clin.

[4] Richards TB, Henley SJ, Puckett MC, Weir HK, Huang B, Tucker TC (2017). Lung cancer survival in the United States by race and stage (2001–2009): findings from the CONCORD-2 study. Cancer.

[5] Sperduto PW, Yang TJ, Beal K, Pan H, Brown PD, Bangdiwala A (2017). Estimating survival in patients with lung cancer and brain metastases: an update of the graded prognostic assessment for lung cancer using molecular markers (Lung-molGPA). JAMA Oncol.

[6] Rahal Z, El Nemr S, Sinjab A, Chami H, Tfayli A, Kadara H (2017). Smoking and lung cancer: a geo-regional perspective. Front Oncol.

[7] Dias M, Linhas R, Campainha S, Conde S, Barroso A (2017). Lung cancer in never-smokers—what are the differences?. Acta Oncol (Stockholm, Sweden).

[8] Oberndorfer F, Müllauer L (2018). Molecular pathology of lung cancer: current status and perspectives. Curr Opin Oncol.

[9] Schrank Z, Chhabra G, Lin L, Iderzorig T, Osude C, Khan N (2018). Current molecular-targeted therapies in NSCLC and their mechanism of resistance. Cancers.

[10] Mender I, LaRanger R, Luitel K, Peyton M, Girard L, Lai TP (2018). Telomerase-mediated strategy for overcoming non-small cell lung cancer targeted therapy and chemotherapy resistance. Neoplasia (New York, NY).

[11] Lin C, Yang L (2018). Long noncoding RNA in cancer: wiring signaling circuitry. Trends Cell Biol.

[12] Farazi TA, Spitzer JI, Morozov P, Tuschl T (2011). miRNAs in human cancer. J Pathol.

[13] Miao W, Li N, Gu B, Yi G, Su Z, Cheng H (2020). LncRNA DLGAP1-AS2 modulates glioma development by up-regulating YAP1 expression. J Biochem.

[14] Jiang L, Zhao Z, Zheng L, Xue L, Zhan Q, Song Y (2017). Downregulation of miR-503 promotes ESCC cell proliferation, migration, and invasion by targeting cyclin D1. Genom Proteomics Bioinform.

[15] Braun DJ, Bachstetter AD, Sudduth TL, Wilcock DM, Watterson DM, Van Eldik LJ (2019). Genetic knockout of myosin light chain kinase (MLCK210) prevents cerebral microhemorrhages and attenuates neuroinflammation in a mouse model of vascular cognitive impairment and dementia. GeroScience.

[16] Matyi S, Jackson J, Garrett K, Deepa SS, Unnikrishnan A (2018). The effect of different levels of dietary restriction on glucose homeostasis and metabolic memory. GeroScience.

[17] Antonia SJ, Villegas A, Daniel D, Vicente D, Murakami S, Hui R (2018). Overall survival with durvalumab after chemoradiotherapy in stage III NSCLC. N Engl J Med.

[18] Navarro-Martin A, Aso S, Cacicedo J, Arnaiz M, Navarro V, Rosales S (2016). Phase II trial of SBRT for stage I NSCLC: survival, local control, and lung function at 36 months. J Thoracic Oncol: Off Publ Int Assoc Study Lung Cancer.

[19] Noguchi T, Toiyama Y, Kitajima T, Imaoka H, Hiro J, Saigusa S (2016). miRNA-503 promotes tumor progression and is associated with early recurrence and poor prognosis in human colorectal cancer. Oncology.

[20] Long J, Ou C, Xia H, Zhu Y, Liu D (2015). MiR-503 inhibited cell proliferation of human breast cancer cells by suppressing CCND1 expression. Tumour Biol: J Int Soc Oncodev Biol Med.

[21] Xu YY, Wu HJ, Ma HD, Xu LP, Huo Y, Yin LR (2013). MicroRNA-503 suppresses proliferation and cell-cycle progression of endometrioid endometrial cancer by negatively regulating cyclin D1. FEBS J.

[22] Yang H, Liu P, Zhang J, Peng X, Lu Z, Yu S (2016). Long noncoding RNA MIR31HG exhibits oncogenic property in pancreatic ductal adenocarcinoma and is negatively regulated by miR-193b. Oncogene.

