# Supplementary figures and images for: LncRNA DLGAP1-AS2 regulates miR-503/cyclin D1 to promote cell proliferation in non-small cell lung cancer
Source: BMC Pulm Med. 2021 Aug 28;21:277. doi: 10.1186/s12890-021-01633-0 (PMC8401159; doi:10.1186/s12890-021-01633-0)

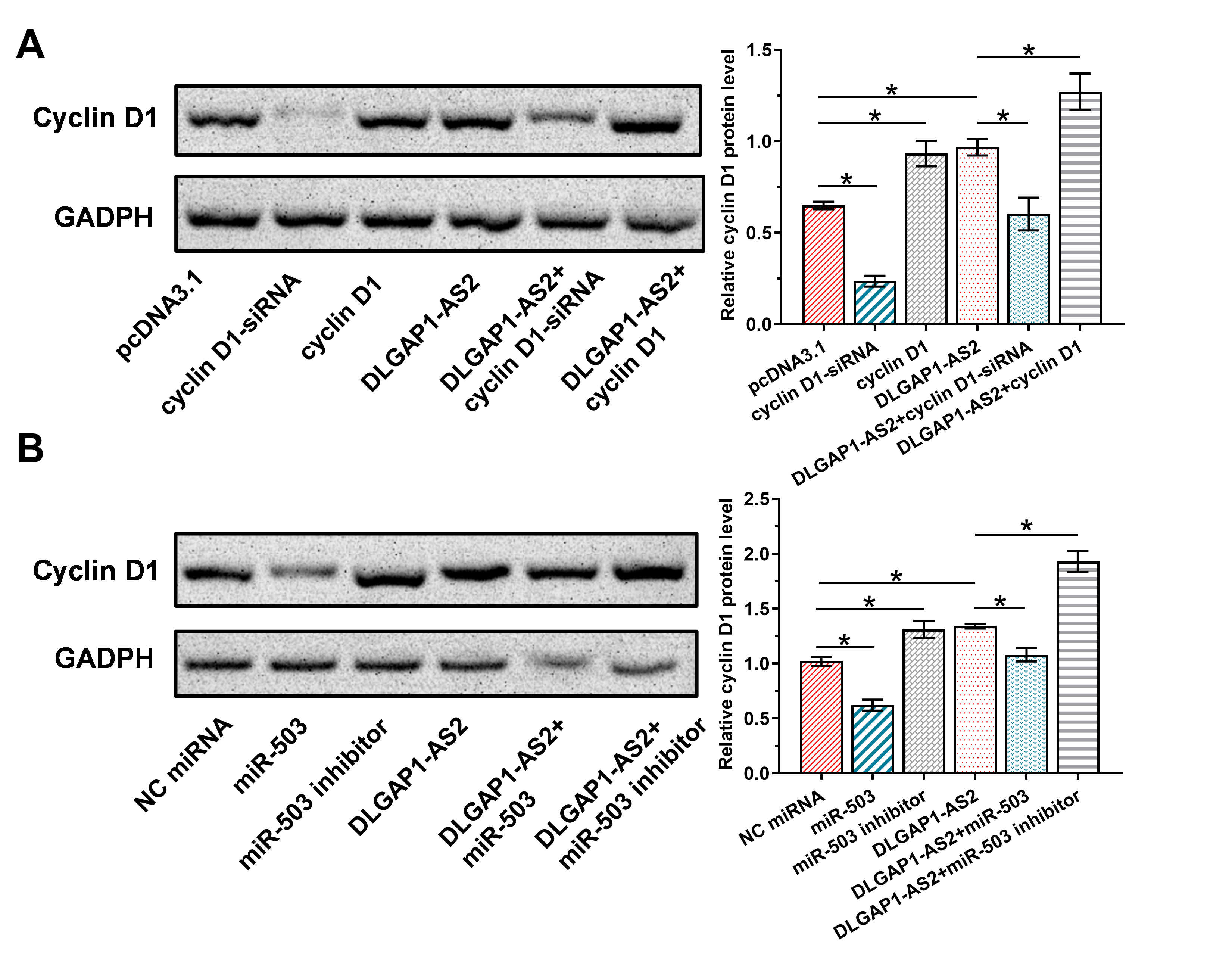

Supplement: Supplementary file 1 — Additional file 1: Figure S1. The relative cyclin D1 protein level in H2170 cells. To further reveal that DLGAP1-AS2 overexpression increased cyclin D1 expression, H2170 cells were transfected with miR-503 inhibitor and cyclin D1-siRNA vectors, and cyclin D1 expression was determined by Western blot. Mean ± SD values of three biological replicates were used to express data of multiple transfection groups. *p < 0.05. [file 12890_2021_1633_MOESM1_ESM.tif]
